# Characteristics and clinical treatment outcomes of chronic hepatitis B children with coexistence of hepatitis B surface antigen (HBsAg) and antibodies to HBsAg

**DOI:** 10.1186/s12916-024-03294-2

**Published:** 2024-02-20

**Authors:** Yingping Gu, Shuangjie Li, Zhenzhen Yao, Xin Lai, Meng Yang, Yi Xu, Songxu Peng

**Affiliations:** 1 Department of Maternal and Child Health, Xiangya School of Public Health, Central South University, No. 172 Tongzipo Road, Yuelu District, Changsha, Hunan 410078 China; 2 Liver Disease Center, Hunan Children’s Hospital, Changsha, 410000 China

**Keywords:** Chronic hepatitis B, Clinical outcomes, HBsAb, Coexistence

## Abstract

**Background:**

The coexistence of hepatitis B surface antigen (HBsAg) and hepatitis B surface antibody (HBsAb) represents an uncommon serological pattern observed in patients with hepatitis B virus (HBV) infection, and its underlying mechanism and clinical significance have not been well established. The aim of this study was to investigate the association between this serological profile and clinical treatment outcomes in children with chronic hepatitis B (CHB).

**Methods:**

This retrospective cohort study included 372 treatment-naïve CHB children from the Hunan Children’s Hospital. The participants were categorized into HBsAb-positive group and HBsAb-negative group. The associations between HBsAb positive status to clinical outcomes were assessed using Cox proportional hazard regression. Receiver operating characteristic curve was conducted to evaluate the prediction ability in HBsAg loss.

**Results:**

The coexistence of HBsAg and HBsAb accounted for 23.39% (87/372) of the participants. The crude incidence rates of HBsAg loss, hepatitis B e antigen (HBeAg) clearance, and HBV-DNA undetectability were higher in the HBsAb-positive group compared with the HBsAb-negative group (37.46 vs. 17.37, 49.51 vs. 28.66, 92.11 vs. 66.54 per 100 person-years, respectively, all *P* < 0.05). The Cox regression analysis revealed a significant association between this serological profile and an increased likelihood of HBsAg loss (HR = 1.78, *P* = 0.001), and HBeAg clearance (HR = 1.78, *P* = 0.001). In addition, a combination of HBsAb ≥ 0.84 log10 IU/L and age ≤ 5 years can help identify patients likely to achieve HBsAg loss after antiviral therapy, with an AUC of 0.71.

**Conclusions:**

Children who are positive for both HBsAg and HBsAb demonstrate a higher probability of favorable outcomes after antiviral treatment. Thus, children with HBsAb-positive CHB should be actively treated to achieve functional cure.

**Supplementary Information:**

The online version contains supplementary material available at 10.1186/s12916-024-03294-2.

## Background

Despite the implementation of universal vaccination programs, hepatitis B virus (HBV) infection of children remains a significant global public health challenge [1–3]. According to the report from WHO in 2015, the prevalence of hepatitis B surface antigen (HBsAg) positivity among children under the age of 5 was estimated to be approximately 1.3% on a global scale [3] and it was estimated to be 0.7% in 2022 [4]. While chronic HBV infection typically follows a benign disease course during childhood, it is noteworthy that a proportion of children (3%-5%) may still experience progression to cirrhosis, and 0.01%-0.03% may develop hepatocellular carcinoma (HCC) in adulthood [5]. Therefore, children with chronic hepatitis B (CHB) require active antiviral therapy to delay the disease progression and minimize the risk of long-term adverse outcomes [6]. The optimum result of antiviral therapy for CHB is commonly considered a functional cure, marked by the absence of HBsAg and undetectable HBV-DNA in the serum, with or without the seroconversion of hepatitis B surface antibodies (HBsAb) [7]. The pursuit of a functional cure should be given high priority for certain select patients.

Serologic patterns, encompassing various serologic markers of HBV, are of utmost importance in the diagnosis and evaluation of CHB. HBsAg is a crucial serologic marker that serves as a significant indicator of ongoing HBV infection. However, HBsAb is an antibody with specific protective properties that can neutralize HBsAg. Consequently, HBsAb positivity often signifies the recovery of HBV infection and the subsequent loss of HBsAg. There has been a growing number of studies on the coexistence of HBsAg and HBsAb in clinical practice since its initial report in 1976 [8–13]. This atypical serological pattern in HBV infection lacks a well-established understanding of its underlying mechanism and clinical significance [12, 14–16]. Several studies have indicated a potential association between this serological profile and significant clinical implications, such as advanced fibrosis, HCC, and liver failure [17–20]. Nevertheless, prior research has primarily concentrated on adult populations, with limited data on children. Furthermore, the impact of the coexistence of HBsAg and HBsAb on antiviral treatment responses remains unknown. Therefore, there is a compelling need to conduct further research in this area.

In the current study, we sought to analyze the characteristics of children with HBsAb-positive CHB. Furthermore, we explored the potential correlations between this serological profile and the clinical outcomes of antiviral treatment, providing an important basis for decision-making in the therapy of CHB.

## Methods

### Study population

This retrospective cohort study included children and adolescents, aged 1–17 years, with treatment-naïve CHB who received antiviral treatment at Hunan Children’s Hospital between June 2016 and April 2023. The diagnosis of CHB adhered to the guidelines on prevention and treatment for chronic hepatitis B (2022 version) [6] including HBsAg positive for over 6 months, persistently or repeatedly abnormal ALT levels, or indications of significant inflammation, necrosis, or significant fibrosis in liver histology. Individuals with nonalcoholic hepatic steatosis or other known causes of chronic liver diseases; coinfection with HCV, HDV, HIV, or cytomegalovirus; concomitant presence of other cancerous neoplasms; insufficient available data; or duration of antiviral treatment of less than 6 months were excluded from the study. Approval for this study was granted by the ethics boards of Xiangya School of Public Health Central South University (XYGW-2023–123). Owing to its retrospective design, there was an exemption from informed consent.

### Definitions and outcomes

The coexistence of HBsAg and HBsAb was determined through simultaneous positivity for those two markers. The participants of the study were categorized into two distinct groups: the HBsAb-positive group consisting of patients who tested positive results for both HBsAg and HBsAb at baseline, and the HBsAb-negative group comprising patients who were HBsAg single-positive at baseline. A positive threshold was established for the HBsAb titer, defined as greater than 10 IU/L (1 log_10_ IU/L). During the follow-up period, regular examinations of virologic and biochemical biomarkers were conducted. The primary outcome was the achievement of HBsAg loss, which was defined as having two consecutive measurements under 0.05 IU/mL. Additional outcomes included HBeAg clearance, which was defined as attaining HBeAg level below 1 COI, and HBV DNA undetectability, defined as achieving DNA level below 100 IU/mL. The follow-up duration was determined by measuring the time elapsed from the initiation of enrollment until the occurrence of endpoints, loss to follow-up, or the concluding follow-up date (April 2023).

### Laboratory testing

Serologic markers for HBV were assessed by Electrochemiluminescence assay (Roche Diagnostics GmbH, Mannheim, Germany). HBV DNA levels and HBV genotype were assessed using the Quantitative Fluorescence Diagnostic HBV Kit and HBV Genotype Kit (Sansure Biotech, Changsha, China), respectively. The alanine aminotransferase (ALT) and aspartate aminotransferase (AST) were measured by the Bayer-2400 automatic biochemistry analyzer. Experienced pathologists, who were unaware of the corresponding clinical data, performed the assessment of liver biopsy samples. The stages of liver fibrosis and grades of inflammation were determined using the Scheuer scoring system, which categorized them into five stages (0–4).

### Statistical analysis

Continuous variables were condensed by presenting mean and standard deviation, or median and interquartile range, based on the data's distribution characteristics. Categorical variables were expressed by frequencies and percentages. For the comparison of different categories of variables, statistical tests such as t-tests, Mann–Whitney U tests for continuous variables and chi-square tests for categorical variables were utilized. The crude rates of clinical outcomes were presented in person-years (PYs). The 95% confidence intervals (CIs) of relative incidence were obtained using Poisson regression. Kaplan–Meier curves were utilized to estimate the cumulative incidence of clinical outcomes, and the log-rank test was employed to compare disparities between the two groups. Cox proportional hazard regression was used to evaluate the associations between the coexistence of HBsAg and HBsAb with clinical treatment outcomes, while subgroup analyses were conducted according to HBV genotype, inflammation grade, and fibrosis stage. Using restricted cubic spline (RCS) plots within Cox proportional hazard models, we examined the associations of HBsAb levels with all concluding events, considering HBsAb as a continuous scale. To assess the predictive effect of HBsAb, receiver operating characteristic curve (ROC) was conducted, with the optimal cutoff value pinpointed via the maximum of the Youden index. The statistical analyses and visualization were conducted utilizing R software (version 4.2.2) and Graphpad Prism (version 9.0), with a significance level of *P* < 0.05.

## Results

### Baseline characteristics of participants

**Fig. 1:**
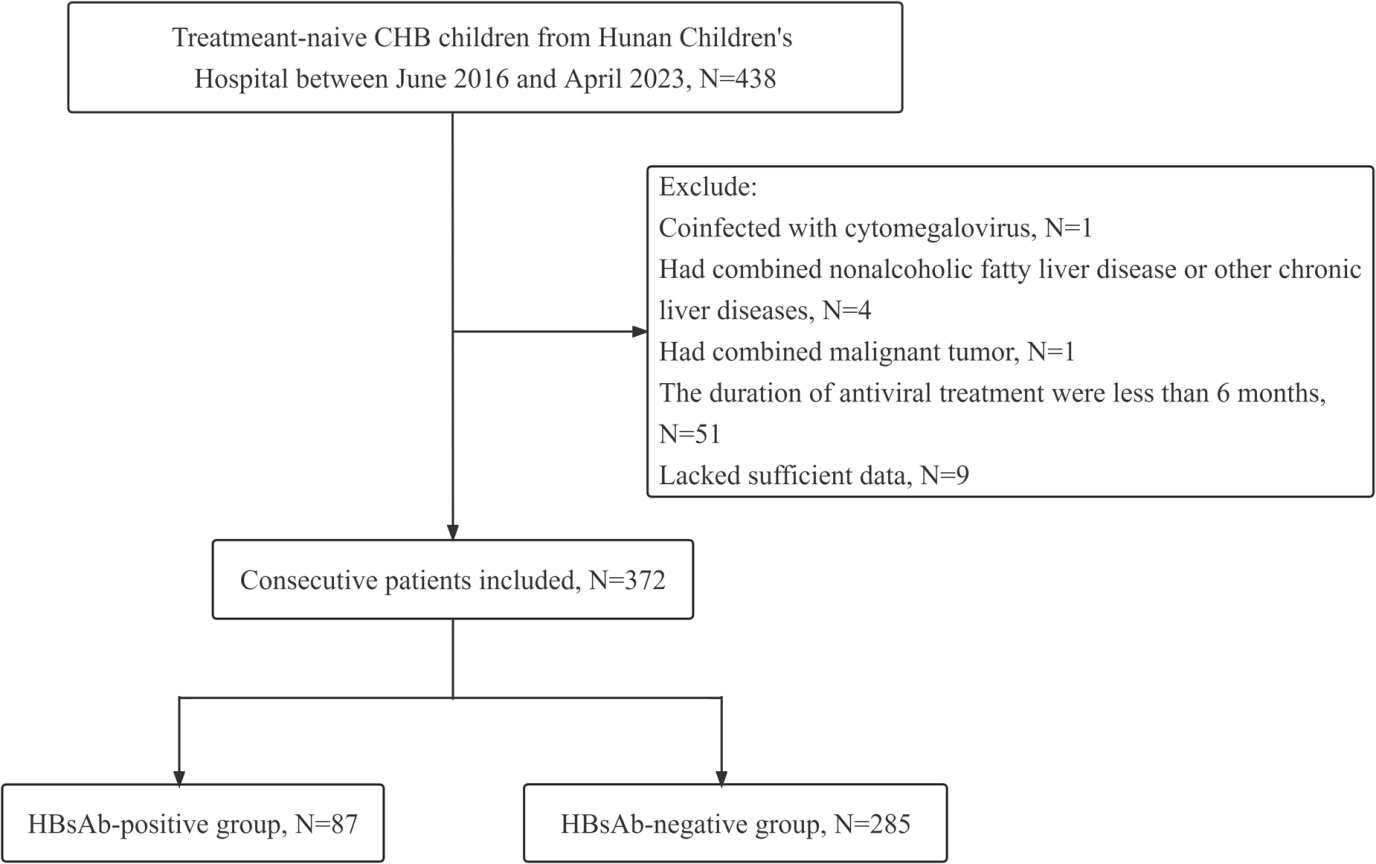
Flow chart of participants selection

Figure 1 illustrates the study enrollment process. A sample size of 372 young participants was encompassed in this study, comprising 231 (62.10%) males and 141 (37.90%) females. Among the total subjects, 87 out of 372 patients (23.39%) were found to be positive for HBsAb. Basic information of participants is presented in Table 1. In brief, no significant disparity was observed in terms of gender, maternal HBV infection status, antiviral treatment regimens, HBV genotype, grade of inflammation, stage of fibrosis, and positive rates of HBeAg between the HBsAb-positive and HBsAb-negative groups. However, the median levels of HBsAg and HBV DNA of the HBsAb-positive group were found to be significantly lower compared to the HBsAb-negative group (3.64 vs. 4.36 log_10_IU/mL, *P* < 0.001; 6.46 vs. 7.24 log_10_IU/mL, *P* = 0.002, respectively). Conversely, the median ALT and AST level of patients in the HBsAb-positive group was higher than that in the HBsAb-negative group (62.20 vs. 39.70 IU/L, *P* < 0.001; 58.20 vs. 46.10 IU/L, *P* = 0.003, respectively). Additionally, compared to the HBsAb-negative group, the patients of the HBsAb-positive group began antiviral treatment at a younger median age(3 vs. 5 years, *P* = 0.003). All patients received antiviral treatment, primarily taking nucleos(t)ide analogue drugs (NAs) combined with interferon/peg-interferon treatment (352/372, 94.62%). No significant difference was observed in the treatment regimens between the two groups. (*P* = 0.135).

**Table 1:** Baseline characteristics of participants

Variables	Total(*n* = 372)	HBsAb-negative group(*n* = 285)	HBsAb-positive group(*n* = 87)	P
Age (years), mean (SD)	4(3,8)	5(3,8)	3(2,6)	**0.003**
Gender, n (%)				0.609
Male	231(62.10)	179(62.81)	52(59.77)	
Female	141(37.90)	106(37.19)	35(40.23)	
Maternal HBV infection status, n (%)				0.569
Yes	307(82.53)	236(82.81)	71(81.61)	
No	14(3.76)	12(4.21)	2(2.30)	
Missing	51(13.71)	37(12.98)	14(16.09)	
HBV Genotype, n (%)				0.518
B	257(69.09)	196(68.77)	61(70.11)	
C	51(13.71)	42(14.74)	9(10.34)	
Missing	64(17.2)	47(16.49)	17(19.55)	
Inflammation grade, n (%)				0.072
0–1	137(36.83)	114(40.00)	23(26.44)	
2–4	89(23.92)	65(22.81)	24(27.59)	
Missing	146(39.27)	106(37.19)	40(45.97)	
Fibrosis stage, n (%)				0.217
0–1	180(48.39)	145(50.88)	35(40.23)	
2–4	46(12.37)	34(11.93)	12(13.79)	
Missing	146(39.27)	106(37.19)	40(45.97)	
HBsAg (log10 IU/ml), med (IQR)	4.25(3.20,4.71)	4.36(3.52,4.72)	3.64(2.44,4.39)	**< 0.001**
HBeAg, n (%)				0.969
Positive	346(93.01)	265(92.98)	81(93.10)	
Negative	26(6.99)	20(7.02)	6(6.90)	
HBV DNA (log_10_ IU/ml), med (IQR)	7.04(5.53,7.91)	7.24(5.58,8.00)	6.46(5.36,7.31)	**0.002**
ALT (IU/L), med (IQR)	43.20(24.20,78.08)	39.70(22.85,68.4)	62.20(32.60,91.30)	**0.001**
AST (IU/L), med (IQR)	48.80(35.25, 78.20)	46.10(32.30,71.35)	58.20(41.20,87.00)	**0.003**
Treatment protocol, n (%)				0.135
NAs	8(2.15)	4(1.40)	4(4.60)	
Interferon/peginterferon	12(3.23)	8(2.81)	4(4.60)	
Combination therapy	352(94.62)	273(95.79)	79(90.80)	

*Abbreviations*: *ALT* Alanine aminotransferase, *AST* Aspartate aminotransferase, *HBeAg* Hepatitis B e antigen, *HBsAb* Antibody against hepatitis B surface antigen, *HBsAg* Hepatitis B surface antigen, *HBV* Hepatitis B virus, *IQR* Interquartile range, *NAs* Nucleos(t)ide analogues, *SD* Standard deviation

By multivariate analysis (Additional file 1: Table S1), our study found that younger age [odds ratio(OR) = 0.92, *P* = 0.028] and lower HBsAg titer (OR = 0.51, *P* < 0.001) were independently associated with HBsAb-positive status at baseline.

### Crude incidence rates and cumulative probabilities of clinical outcomes

Table 2 presents the crude incidence rates of clinical outcomes, including seroclearance of HBsAg and HBeAg, as well as the undetectability of HBV DNA. During the 407.95 PYs of follow-up, the crude incidence rate of DNA undetectability was 71.58 per 100 PY, which was more common and seemed to occur more rapidly than HBsAg loss (21.40 per 100 PY, 719.93 PYs) and HBeAg seroclearance (32.88 per 100 PY, 568.73 PYs). At the end of the follow-up, 154 patients achieved HBsAg loss, including 54(37.46 per 100 PY) from the HBsAb-positive group and 100(17.37 per 100 PY) from the HBsAb-negative group [hazard ratio (HR) = 2.16; 95% CI: 1.52–3.03]. The crude incidence rates of HBeAg seroclearance and HBV DNA undetectability between the HBsAb-positive and HBsAb-negative groups were 49.51 vs. 28.66 per 100 PY (HR = 1.73; 95% CI: 1.24–2.38) and 92.11 vs. 66.54 per 100 PY (HR = 1.38; 95% CI: 1.05–1.81), respectively. The differences reached statistical significance.

**Table 2:** Crude incidence rate and HR (95%CI) of clinical outcomes according to HBsAb status

Clinical outcomes	Metrics	Total	HBsAb-negative group	HBsAb-positive group	Unadjusted model		Adjusted model	
					HR (95% CI)	P	HR (95% CI)	P
HBsAg loss	PYs of follow-up	719.93	575.76	144.17	2.21(1.59–3.08)	**< 0.001**	1.78(1.29–2.48)	**0.001**
	No. of events	154	100	54				
	Crude incidence rate (per 100 PY)	21.40	17.37	37.46				
	HR (95% CI)	-	1(Ref)	2.16(1.52–3.03)				
HBeAg clearance	PYs of follow-up	568.73	453.60	115.13	1.85(1.35–2.53)	**< 0.001**	1.78(1.25–2.53)	**0.001**
	No. of events	187	130	57				
	Crude incidence rate (per 100 PY)	32.88	28.66	49.51				
	HR (95% CI)	-	1(Ref)	1.73(1.24–2.38)				
HBV DNA undetectability	PYs of follow-up	407.95	327.61	80.34	1.51(1.15–1.96)	**0.003**	1.27(0.95–1.69)	0.104
	No. of events	292	218	74				
	Crude incidence rate (per 100 PY)	71.58	66.54	92.11				
	HR (95% CI)	-	1(Ref)	1.38(1.05–1.81)				

Adjusted model: Adjusted for age, gender, treatment protocol, baseline HBsAg, HBeAg, HBV DNA, ALT, and AST

*Abbreviations*: *CI* Confidence interval, *HBeAg* Hepatitis B e antigen, *HBsAb* Antibody against hepatitis B surface antigen, *HBsAg* Hepatitis B surface antigen, *HBV* Hepatitis B virus, *HR* Hazard ratio, *PY* Person-years

**Fig. 2:**
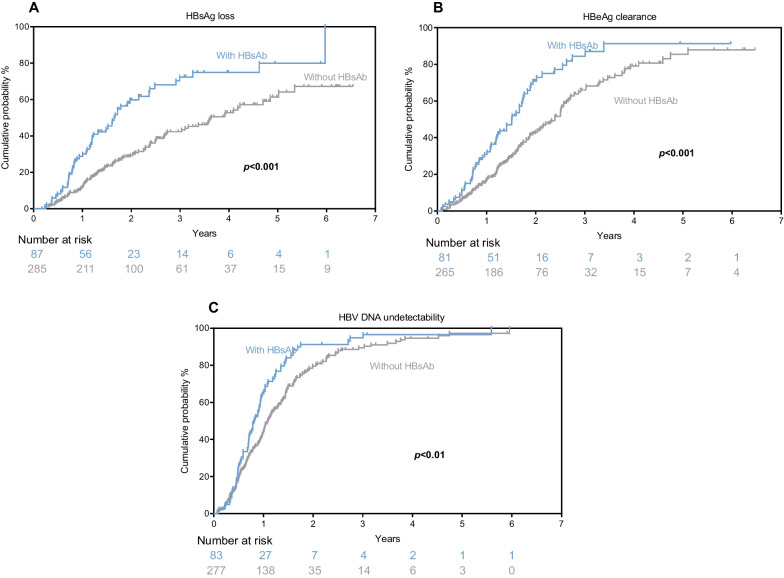
Kaplan–Meier curves of treatment outcomes between HBsAb-positive group and HBsAb-negative group

Figure 2 shows the cumulative incidence rates of clinical outcomes. Based on Kaplan–Meier analysis, the cumulative probabilities of various clinical treatment outcomes during the follow-up period were found to be higher in the HBsAb-positive group compared to the HBsAb-negative group (all *P* < 0.05, log-rank test).

In addition, for children with abnormal ALT levels at baseline, we analyzed their ALT normalization after antiviral treatment. Our results revealed that there was no significant difference in the crude incidence rate (Additional file 1: Table S2) and cumulative incidence rate (Additional file 1: Figure S1) of ALT normalization between the HBsAb-positive group and the HBsAb-negative group.

### Baseline HBsAb status associated with clinical outcomes

The HRs and 95% CIs of clinical outcomes based on the status of HBsAb are presented in Table 2. Compared to the HBsAb-positive group, the patients in the HBsAb-negative group had a 1.78-fold higher probability of HBsAg loss after adjusting for potential confounding variables (Adjusted model; HR = 1.78; 95%CI: 1.29–2.48). Similarly, the HBsAb-positive group had a higher probability of HBeAg clearance (Adjusted model; HR = 1.78; 95%CI: 1.25–2.53). However, no association was observed in terms of DNA undetectability (Adjusted model; HR = 1.27; 95% CI: 0.95–1.69).

**Fig. 3:**
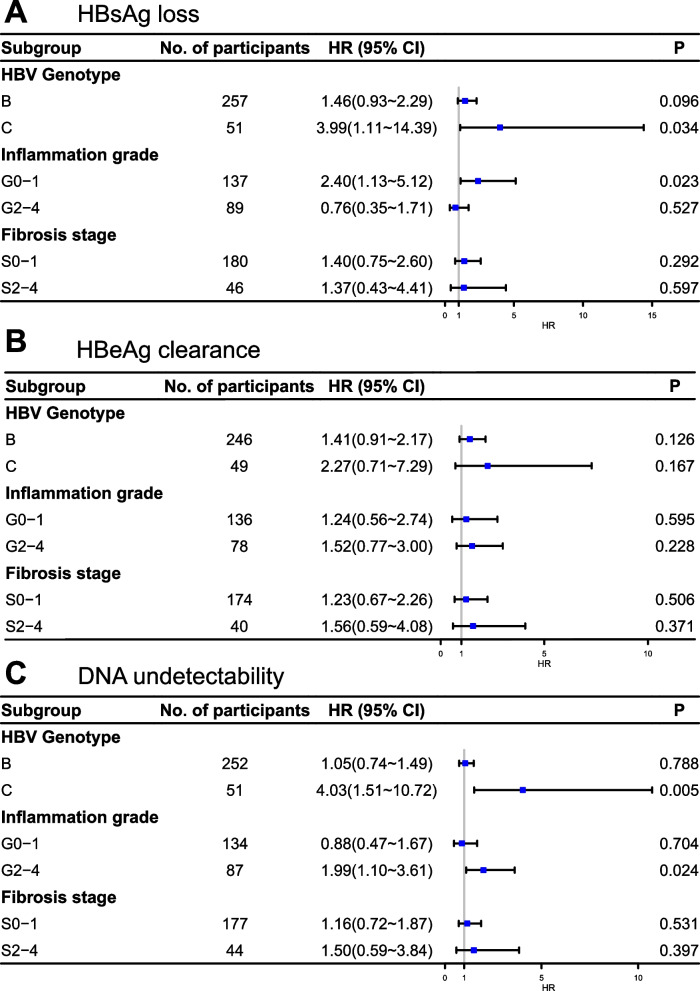
Subgroup analyses of the HR (95% CI) of treatment outcomes according to HBsAb status. The models were adjusted for age, gender, baseline HBsAg, HBeAg, HBV DNA, ALT, and AST

Next, subgroup analyses were conducted to examine whether these associations were consistent across different subgroups, including HBV genotypes, inflammation grades, and fibrosis stages. According to the results depicted in Fig. 3, HBsAb positivity was independently linked to HBsAg loss in patients carrying genotype C and inflammation grades 0–1 (HR = 3.99, 95%CI: 1.11–14.39; HR = 2.40, 95% CI: 1.13–5.12). In patients with genotype C and inflammation grades 2–4, HBsAb positivity was independently related to DNA undetectability (HR = 4.03, 95%CI:1.51–10.72; HR = 1.99, 95% CI: 1.10–3.61, respectively). No association was found between HBsAb status and HBeAg clearance across all subgroups.

### Dose–response relationship between HBsAb level and treatment outcomes

**Fig. 4:**
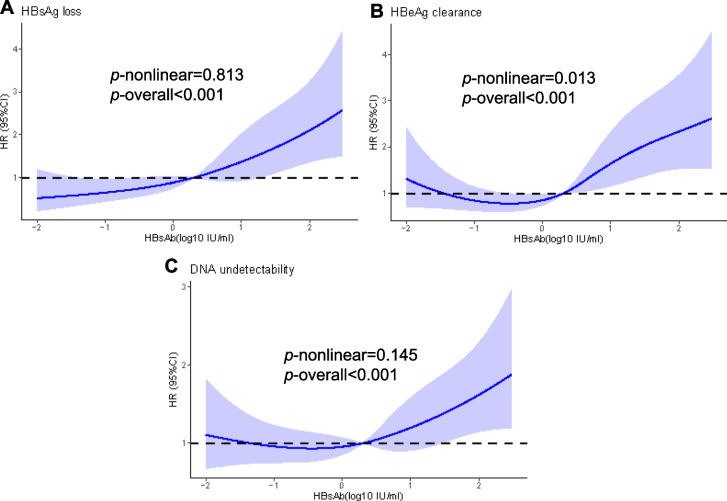
The restricted cubic spline of the association between HBsAb level and the risk of treatment outcomes. The models were adjusted for age, gender, treatment protocol, baseline HBsAg, HBeAg, DNA, ALT, and AST. Curves showed HRs compared with the chosen reference HBsAb level of 0.30 log_10_IU/L (2 IU/L)

The RCS analysis (Fig. 4) demonstrated dose–response relationships between the continuous change of HBsAb levels and the probabilities of clinical treatment outcomes among children with CHB. The results revealed a linear dose–response relationship between HBsAb levels with HBsAg loss (P-nonlinear = 0.813, P-overall < 0.001) and DNA undetectability (P-nonlinear = 0.145, P-overall < 0.001), after adjusting for other covariates. As HBsAb levels increased at the beginning of treatment, the probabilities of HBsAg loss and DNA undetectability also increased accordingly. Additionally, the analysis results revealed a "J-shaped" correlation between HBsAb levels and the probability of HBeAg clearance with a threshold at -0.49 log_10_ IU/L (P-nonlinear = 0.013, P-overall < 0.001).

### Predictive ability for HBsAg loss

**Fig. 5:**
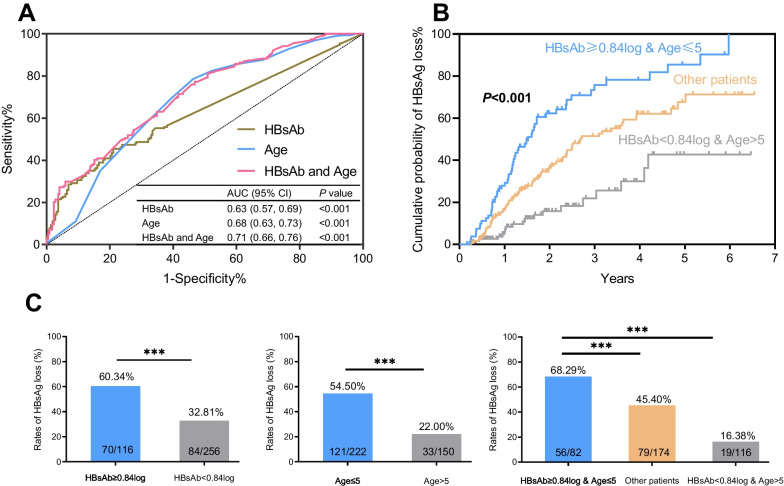
Predictive effect of HBsAb and age on HBsAg loss. **A** Receiver operating characteristic curve for HBsAg loss. **B** Kaplan–Meier curves for HBsAg loss according to HBsAb and age at baseline. **C** Comparison of HBsAg loss rates according to HBsAb and age at baseline. *p < 0.05, **p < 0.01, ***p < 0.001

The results of ROC curve analysis (Fig. 5A) demonstrated an area under the curve (AUC) of 0.63 (95% CI: 0.57–0.69, *P* < 0.001) for predicting HBsAg loss using HBsAb titers. When further combined with age, the AUC improved to 0.71 (0.66–0.76, *P* < 0.001) with a sensitivity of 0.77 and specificity of 0.47. The optimal cutoff values for HBsAb titers and age in predicting HBsAg loss were determined to be 0.84 log_10_ IU/L and 5 years, respectively. As shown in Fig. 5B, the patients (82/372) with HBsAb ≥ 0.84 log_10_ IU/L and age ≤ 5 exhibited the highest cumulative probability of HBsAg loss (*P* < 0.001). Furthermore, Fig. 5C shows that the positive predictive values (PPV) for predicting HBsAg loss based on HBsAb ≥ 0.84 log_10_ IU/L or age ≤ 5 years were 60.34% and 54.50%, respectively. The combination of those two indicators yielded a higher PPV of 68.29%.

## Discussion

In this study, 23.39% (87/372) of children with CHB were tested positive for HBsAb. In addition, these children demonstrated higher rates of achieving favorable outcomes, including seroclearance of both HBsAg and HBeAg, as well as the undetectability of HBV DNA, compared to those children who are negative for HBsAb. Importantly, the strength of the association increased with higher levels of HBsAb.

Despite it has been about half a century since this serological pattern was first reported [8], the molecular mechanisms underlying this phenomenon are still unclear. The current mainstream hypotheses included mutations in the viral genome and hosts immune status [12, 15]. Previously reported rates of coexistence of HBsAg and HBsAb vary widely, ranging from 0.3% to over 30% [10, 11, 15, 18, 21, 22]. The coexistence of HBsAg and HBsAb (23.39%, 87/372) in our study was comparable with the incidence (20.67%, 234/1132) reported by Wang et al. [23]. However, the incidence of coexistent HBsAg and HBsAb among children with chronic HBV infection was only 0.81% (1/124) in North America [24], which is much lower than our result. A large real-world study from China revealed that the occurrence rate was 4.24% (277/6534) among treatment-naive adults with CHB [20]. Additionally, another study from China indicated a prevalence of 47.30% (205/433) for this phenomenon in the population with CHB [18] Possible reasons for this difference in incidence include heterogeneity of study populations, different disease phases and the method of detection [25–27].

The analysis of clinical characteristics in our study revealed that children with HBsAb-positive CHB had lower levels of HBsAg and HBV DNA, which aligns with results from previous studies [28, 29]. The possible explanation is that HBsAb could neutralize HBsAg, thereby reducing the levels of HBsAg. Moreover, the generation of HBsAb signifies a certain degree of immune response within the host, leading to a reduction in the levels of viral DNA [28, 30]. Additionally, these children with HBsAb displayed elevated levels of ALT and AST, which may be attributed to a more pronounced immune response against HBV due to the presence of HBsAb. Because the vigorous immune response usually leads to extensive liver inflammation and subsequent hepatocellular damage, thereby contributing to increased ALT and AST levels.

Previous studies demonstrated that HBsAb may promote clearance of HBV and achieve functional cure via multiple mechanisms [31]. Our results further support these findings, as we observed significantly higher incidence rates of HBsAg loss, HBeAg seroclearnace, and undetectable HBV DNA among children with HBsAb. This observation aligns with a Korean study that showed that individuals with this serological pattern exhibited a higher rate of HBsAg clearance compared to those without HBsAb [29]. Similarly, some reports documented that patients with coexisting of HBsAg and HBsAb had higher probabilities to achieve clearance of HBsAg, HBeAg, and HBV DNA after treatment with NAs or interferon [32, 33]. Furthermore, it has been widely reported that baseline higher ALT levels, lower levels of HBV DNA and HBsAg are predictive indicators for the effectiveness of antiviral therapy [34, 35]. This may partly explains the increased clearance rates of HBsAg and HBeAg in children with coexistence of HBsAg and HBsAb. Moreover, the phenomenon that children with HBsAb-positive CHB were more likely to attain HBsAg loss seemed to hint the coexistence of HBsAg and HBsAb may be an intermediate transition in the clearance of HBsAg.

The AUC of the prediction model was estimated to be 0.63 when we used HBsAb level to predict HBsAg loss. Combining with age, the value of AUC improved to 0.71. A combination of HBsAb ≥ 0.84 log_10_ IU/L and age ≤ 5 can help clinical physicians identify patients likely to achieve HBsAg loss after antiviral therapy. This suggests that younger patients who had relatively high levels of HBsAb have a greater likelihood of achieving HBsAg loss, which is a key milestone in achieving the goal of hepatitis B elimination. Therefore, timely diagnosis and early initiation of antiviral treatment in pediatric patients with high HBsAb levels can significantly improve treatment outcomes.

Our study deserves attention as this research marks the inaugural investigation into the association between the coexistence of HBsAg and HBsAb and clinical treatment outcomes in children with CHB. Inevitably, the current study has several limitations. Firstly, although this study investigated the longitudinal outcomes of children with HBsAb, the underlying mechanism explaining the increased rates of favorable outcomes remains unclear. Further research is needed to fully elucidate this. Secondly, age may serve as a confounding factor, although it has been appropriately adjusted in multivariate analysis, which may lead to selection bias. Thirdly, this study was conducted at a single hospital. The generalizability of the findings to different clinical settings may be limited. Therefore, to validate the results and enhance the external validity of the findings, it is recommended to conduct multicenter studies with larger sample sizes.

## Conclusions

Our study suggested that children with HBsAb-positive CHB demonstrated a higher probability of favorable outcomes. Moreover, HBsAb is an independent predictor of HBsAg loss and HBeAg clearance. Therefore, pediatric patients with this serological pattern should be actively treated, considering the higher probability of achieving a functional cure.

### Supplementary Information

**Additional file 1:** **Table S1.** Multivariate analysis of HBsAb-positive status at baseline. **Table S2.** Crude incidence rate (per 100 PY) of ALT normalization. **Figure S1.** Kaplan-Meier curves of ALT normalization between HBsAb-positive group and HBsAb-negative group.

## Data Availability

The datasets generated during and/or analysed during the current study are not publicly available but are available from the corresponding author on reasonable request.

## Abbreviations

ALT: Alanine aminotransferase
AST: Aspartate aminotransferase
AUC: Area under curve
CHB: Chronic hepatitis B
CI: Confidence interval
HBeAg: Hepatitis B e antigen
HBsAb: Antibody against hepatitis B surface antigen
HBsAg: Hepatitis B surface antigen
HBV: Hepatitis B virus
HCC: Hepatocellular carcinoma
HR: Hazard ratio
NAs: Nucleos(t)ide analogues
OR: Odds ratio
PPV: Positive predictive value
PY: Person-years
RCS: Restricted cubic spline
ROC: Receiver operating characteristic curve
