# Supplementary material for: Characteristics and clinical treatment outcomes of chronic hepatitis B children with coexistence of hepatitis B surface antigen (HBsAg) and antibodies to HBsAg
Source: BMC Med. 2024 Feb 20;22:77. doi: 10.1186/s12916-024-03294-2 (PMC10877877; doi:10.1186/s12916-024-03294-2)

**Additional file 1**

**Supplementary Tables and Figures Legends**

Table S1. Multivariate analysis of HBsAb-positive status at baseline.

Table S2. Crude incidence rate (per 100 PY) of ALT normalization.

Figure S1. Kaplan-Meier curves of ALT normalization between HBsAb-positive group and HBsAb-negative group.

Table S1 Multivariate analysis of HBsAb-positive status at baseline.

| Variables | OR (95% CI) | *P* |
| --- | --- | --- |
| Age | 0.92(0.85-0.99) | 0.028 |
| Gender | 1.17(0.69-1.97) | 0.559 |
| ALT | 1.00(0.99-1.01) | 0.613 |
| AST | 1.00(0.99-1.01) | 0.526 |
| HBsAg | 0.51(0.38-0.69) | <0.001 |
| HBeAg | 2.50(0.82-7.58) | 0.107 |
| HBV DNA | 1.04(0.86-1.25) | 0.712 |

Abbreviations: ALT, alanine aminotransferase; AST, aspartate aminotransferase; HBeAg, hepatitis B e antigen; HBsAb, antibody against hepatitis B surface antigen; HBsAg, hepatitis B surface antigen.

Table S2 Crude incidence rate (per 100 PY) of ALT normalization.

| Clinical outcomes | Metrics | Total(n=308) | HBsAb-negative group(n=226) | HBsAb-positive group(n=82) |
| --- | --- | --- | --- | --- |
| ALT normalization | PYs of follow-up | 413.88 | 304.05 | 109.84 |
|  | No. of events | 207 | 142 | 65 |
|  | Crude incidence rate (per 100 PY） | 50.01 | 46.70 | 59.18 |
|  | HR (95% CI) | - | 1(Ref) | 1.27(0.93-1.71) |

NOTE: The upper limit of normal ALT has been redefined as 30 IU/L for male children and 24 IU/L for female children. ALT normalization was defined as observing ALT levels below the upper limit of normal for at least 6 months.

Abbreviations: CI, confidence interval; HBsAb, antibody against hepatitis B surface antigen; HBsAg, hepatitis B surface antigen; HR, hazard ratio; PY, person-years.

Figure S1 Kaplan-Meier curves of ALT normalization between HBsAb-positive group and HBsAb-negative group.


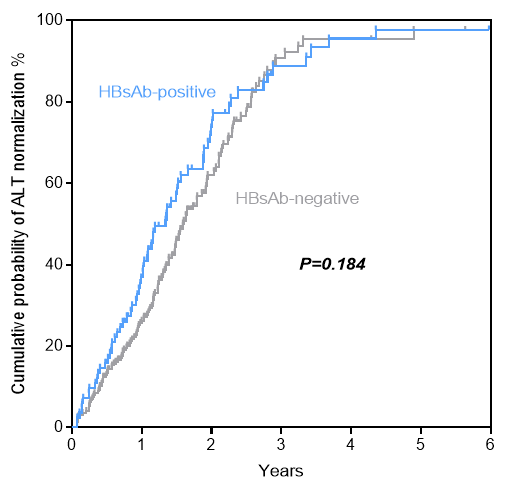

Supplement: Supplementary file 1 — Additional file 1: Table S1. Multivariate analysis of HBsAb-positive status at baseline. Table S2. Crude incidence rate (per 100 PY) of ALT normalization. Figure S1. Kaplan-Meier curves of ALT normalization between HBsAb-positive group and HBsAb-negative group. [file 12916_2024_3294_MOESM1_ESM.docx]
